# Normative optical coherence tomography-based measurements and classification of optic nerve head parameters in adult Congolese subjects

**DOI:** 10.3389/fopht.2026.1687212

**Published:** 2026-01-22

**Authors:** Elke N. Balayi, Nelly N. Kabedi, Doudou Ngwanza, Joseph-Theodore Kelekele, Jean-Claude Mwanza

**Affiliations:** 1 Department of Ophthalmology, University of Kinshasa School of Medicine, Kinshasa, Democratic Republic of Congo; 2 Department of Ophthalmology, University of North Carolina School of Medicine, Chapel Hill, NC, United States

**Keywords:** adult, Congolese, normative data, optic nerve head, optical coherence tomography

## Abstract

**Background:**

Normative data on optic nerve head (ONH) topographic measurements are scarce among black people residing in Africa. Such measurements obtained with imaging modalities such as optical coherence tomography (OCT) are crucial for the diagnosis and management of optic nerve diseases, particularly glaucoma. They assist clinicians in identifying deviations that may indicate disease or abnormalities. The aim of this study was to determine normal reference values of ONH topographic measurements in a Congolese adult population using OCT.

**Methods:**

Hospital-based cross-sectional observational study of 263 healthy adult subjects (18–76 years) who were scanned with a Topcon 3D OCT-2000 device using the optic disc scan pattern. The 2.5^th^, 5^th^, median, 95^th^, and 97.5^th^ percentiles, and the mean of the optic disc area (ODA), optic disc vertical diameter (ODVD), optic disc horizontal diameter (ODHD), vertical cup-to-disc ratio (VCDR), area cup-to-disc ratio (ACDR), optic disc cup area (ODCA), and optic disc rim area (ODRA) were determined.

**Results:**

The median (IQR) was 2.70 (0.92) mm^2^ for ODA, 2.03 (0.32) mm for ODVD, 1.70 (0.5) mm for ODHD, 0.57 (0.20) for VCDR, 0.89 (0.81) mm^2^ for ODCA, 1.74 (0.75) mm^2^ for ODRA. The lower and upper limits of normality were 1.88 and 4.67 mm^2^ (ODA), 1.64 and 2.64 mm (ODVD), 0.00 and 0.81 (VCDR), 0.00 and 2.60 mm^2^ (ODCA), and 1.63 and 3.22 mm^2^ (ODRA). For OCT classification, the range of 2.02-4.19 mm^2^ was considered normal for ODA, 1.73-2.51 mm for ODVD, 0.23-0.76 for VCDR, 0.04-0.63 for ACDR, 0.11-2.33 mm^2^ for ODCA, and 1.69-3.01 mm^2^ for ODRA. ODRA was significantly larger in women than men. None of the optic disc parameters correlated with age.

**Conclusion:**

This study provides population-specific normative data describing ONH morphology in healthy Congolese adults, addressing a critical gap for African populations that remain underrepresented in ocular imaging research. These ONH measurements were significantly greater than found in other populations. They may be used for diagnostic reliability and classification of Congolese subjects.

## 1 Introduction

Evaluation of the optic nerve head (ONH) is an important part of the overall ophthalmic examination. It includes assessment of the optic disc’s location, morphology, size, insertion, coloration, margin sharpness, neuroretinal rim, the presence or absence of peripapillary atrophy and optic disc hemorrhage, estimation of the cup-to-disc ratios (CDR), and the presence of any congenital abnormality. Optic disc topographic measurements are of paramount importance for the diagnosis of glaucoma and other optic nerve abnormalities such as megalopapilla and optic nerve hypoplasia. Assessment of changes to the optic disc is usually the first clinical step in detecting glaucomatous changes. Ascertaining that an optic disc is glaucomatous can be challenging when changes are still minimal and clinically indiscernible. This challenge is compounded by inter-individual and interracial variability in optic disc size within normal populations (1–3) and substantial overlap in measurements such as the vertical cup-to-disc ratio (VCDR) between healthy and glaucomatous discs (4, 5).

In practice, fundus biomicroscopy through a +78D or +90D lens, or indirect ophthalmoscopy through a +20D or +28D lens are used to estimate optic disc measurements such the CDR. In optic disc reading centers, CDRs are estimated on optic disc photographs (6). However, these methods suffer from subjectivity and suboptimal inter-examiner agreement. Spectral domain optical coherence tomography (OCT) has become one of the major methods of choice for quantitative evaluation of the optic disc because it is easy to use, it is patient-friendly, and it provides objective, reproductible, and accurate measurements. Due to interracial and interethnic variations of optic disc measurements, it is generally recommended for each population to generate its own normative values for reliable interpretation of clinical observations and discrimination between normal and pathologic data. In sub-Saharan Africa, OCT-based ONH normative values have been determined among Ethiopians (7), Gabonese (8), Ghanaians (9, 10), and Nigerians (11). In the Democratic Republic of Congo (DRC), one study reported ONH reference measurements acquired with the Heidelberg Retina Tomograph (HRT) (12). However, the HRT has long been phased out because it was not fully automated and time-consuming as it required manually marking the disc and cup margins, leading to measurement variability. In addition, unlike OCT, the HRT did not use the true anatomical landmarks (e.g. Bruch’s membrane opening, internal limiting membrane) to determine the disc and cup margins (13–15), resulting in overestimation of optic disc measurements (16). The purpose of this study was to determine normal reference values of ONH topographic measurements in a normal Congolese adult population using OCT.

## 2 Methods

### 2.1 Study participants

Study participants were recruited among medical and administrative staff, as well as patients attending the eye clinic of the University Hospital of Kinshasa. Requirements to be included in the study were being an 18 years or older person willing to provide a written informed consent, with a normal ocular examination including a best corrected visual acuity greater than or equals to 6/10, refraction between -6 and +3 spherical or between +3 and -3 cylindric diopters, intraocular pressure <21 mmHg, clear media, normal-looking optic disc (intact neuroretinal rim, no pallor or hemorrhage, and no vertical CDR asymmetry of greater than 0.2 between both eyes). Exclusion criteria included media opacities; a history of retinal disease, glaucoma or glaucoma suspicion, uveitis, non-glaucomatous optic nerve disease, systemic disease known to affect the optic nerve and/or the retina, neurological disease, previous neurosurgery, vitreoretinal surgery or laser therapy, treatment with ocular or systemic anti-vascular endothelial growth factor agents, IOP-lowering treatment, topical or systemic steroid therapy, or chemotherapy. The study received approval from the Ethics Committee of the Kinshasa School of Public Health. All participants provided a written informed consent, and the study complied with the Tenets of the Declaration of Helsinki.

### 2.2 Ophthalmologic examination and optical coherence tomography

All participants underwent a standard eye examination that included Snellen visual acuity measurement at 5 meters, autorefractometry (i-Tronix AR-10, Matronix Optotechnik Private Limited, New Delhi, India), slit lamp biomicroscopic examination before and after pupil dilation, Goldman aplanation tonometry, and dilated ophthalmoscopy. Standard Automated Perimetry (Humphrey Visual Field, Carl Zeiss Meditec, Inc., Dublin, CA, USA) was only performed where needed based on clinical findings. Both eyes of each participant were scanned after pupil dilation with a Topcon 3D OCT-2000 device (Topcon Co., Tokyo, Japan) using the optic disc cube 200x200 protocol. All scans were acquired by the same experienced operator (NNK). The scans were quality-controlled for overt segmentation errors and artifacts due to saccades, blinking, and floaters. Only good quality scans (well-centered, quality index ≥60, absence of any artifacts and segmentation failure) were included in the analysis. ONH measurements were generated automatically by the device algorithm. The following parameters were included in this study: optic disc area (ODA, mm^2^), optic disc cup area (ODCA, mm^2^), optic disc vertical diameter (ODVD, mm), optic disc horizontal diameter (ODHD, mm), average cup-to-disc area ratio (ACDR), vertical cup-to-disc ratio (VCDR), and optic disc rim area (ODRA, mm^2^).

### 2.3 Statistical analysis

All analyses were conducted in SPSS version 29.0 (SPSS Boston, USA). Normality of data distribution was evaluated with the Kolmogorov-Smirnov test. Descriptive statistics were reported as median and interquartile along with the 2.5^th^, 5^th^, 95^th^, and 97.5^th^ percentiles for quantitative measures and frequency with proportion for categorical variables. The 2.5^th^ and 97.5^th^ percentiles were designated as the lower and upper limits of normality, respectively, per the International Society for Geographic and Epidemiologic Ophthalmology (ISGEO) (17). The 5^th^ and 95^th^ percentiles were determined to allow stratification based on OCT color-coded classification relative to normal age-matched individuals: green for measurements that fall within 90% (5% to 95%) of the measurements of age-matched normal individuals; orange for measurements outside normal range that fall below 5% of the measurements; and red for measurements greater than 95% of the measurements. The Mann-Whitney U test and the Wilcoxon signed-rank test were used to compare ONH measures between sexes and between fellow eyes, respectively. Comparison of measures between age groups was made using the Kruskal-Wallis’s test. The relationship of ONH parameters with age, sex, BMI, spherical equivalent, and IOP was assessed using the Spearman-rank correlation coefficient. A p-value <0.05 was considered statistically significant for all statistical analyses.

## 3 Results

### 3.1 Characteristics of the study participants

Of the 271 normal subjects who were examined, 8 were subsequently excluded for optic disc margin or cup margin segmentation errors. The general characteristics of the remainder 263 subjects, 54.8% of whom were women are shown in **Table 1**. The median (IQR) age was 36.1 (22.0) years, without significant difference between men and women. Median values were 167 (0.13) cm for height, 68.0 (18.0) kg for weight, 23.9 (5.85) kg/m^2^ for BMI, 14.0 (4.0) mmHg for IOP in right eye, 13.0 (4.0) mmHg for IOP the left eye, and 0.00 (1.12) diopter for ESR in right eye and 0.00 (1.41) diopter for ESR in the left eye. Men were significantly taller and heavier (both p <0.001) but had significantly lower ESR in both eyes than women.

**Table 1:** Characteristics of study participants.

Characteristics	All	Men	Women	p
N (%)	263 (100)	119 (45.2)	144 (54.8)	0.028
18–29 years, n (%)	76 (28.9)	28 (36.8)	48 (63.2)	0.001
30–39 years, n (%)	65 (24.7)	31 (47.7)	34 (52.3)	0.60
40–49 years, n (%)	63 (24.0)	25 (39.7)	38 (60.3)	0.02
≥ 50 years, n (%)	59 (22.4)	26 (44.1)	33 (55.9)	0.20
Mean age ± SD	37.9 ± 13.5	38.7 ± 13.3	37.4 ± 13.7	0.76
Median age (IQR)	36.1 (22.0)	36.3 (22.3)	36.2 (22.4)	0.35
Median height (IQR)	1.67 (0.13)	1.75(0.13)	1.65 (0.08)	<0.001
Median weight (IQR)	68.0 (18.0)	70.0 (16.5)	65.0 (16.0)	<0.001
Median BMI (IQR)	23.9 (5.85)	23.9 (5.9)	23.9 (5.9)	0.76
Median OD IOP (IQR)	14.0 (4.0)	13.0 (4.0)	14.0 (4.0)	0.72
Median OS IOP (IQR)	13.0 (4.0)	13.0 (4.0)	14.0 (4.0)	0.64
Median OD SER (IQR)	0.00 (1.12)	0.00 (1.13)	0.25 (1.22)	0.013
Median OS SER (IQR)	0.00 (1.41)	0.00 (1.59)	0.13 (1.09)	0.016

SD, standard deviation; IQR, interquartile range; IOP, intraocular pressure; SER, spherical equivalent refraction; BMI, body mass index; OD, right eye; OS, left eye.

### 3.2 Normal distribution and OCT-based classification of optic nerve head measurements

**Figure 1:**
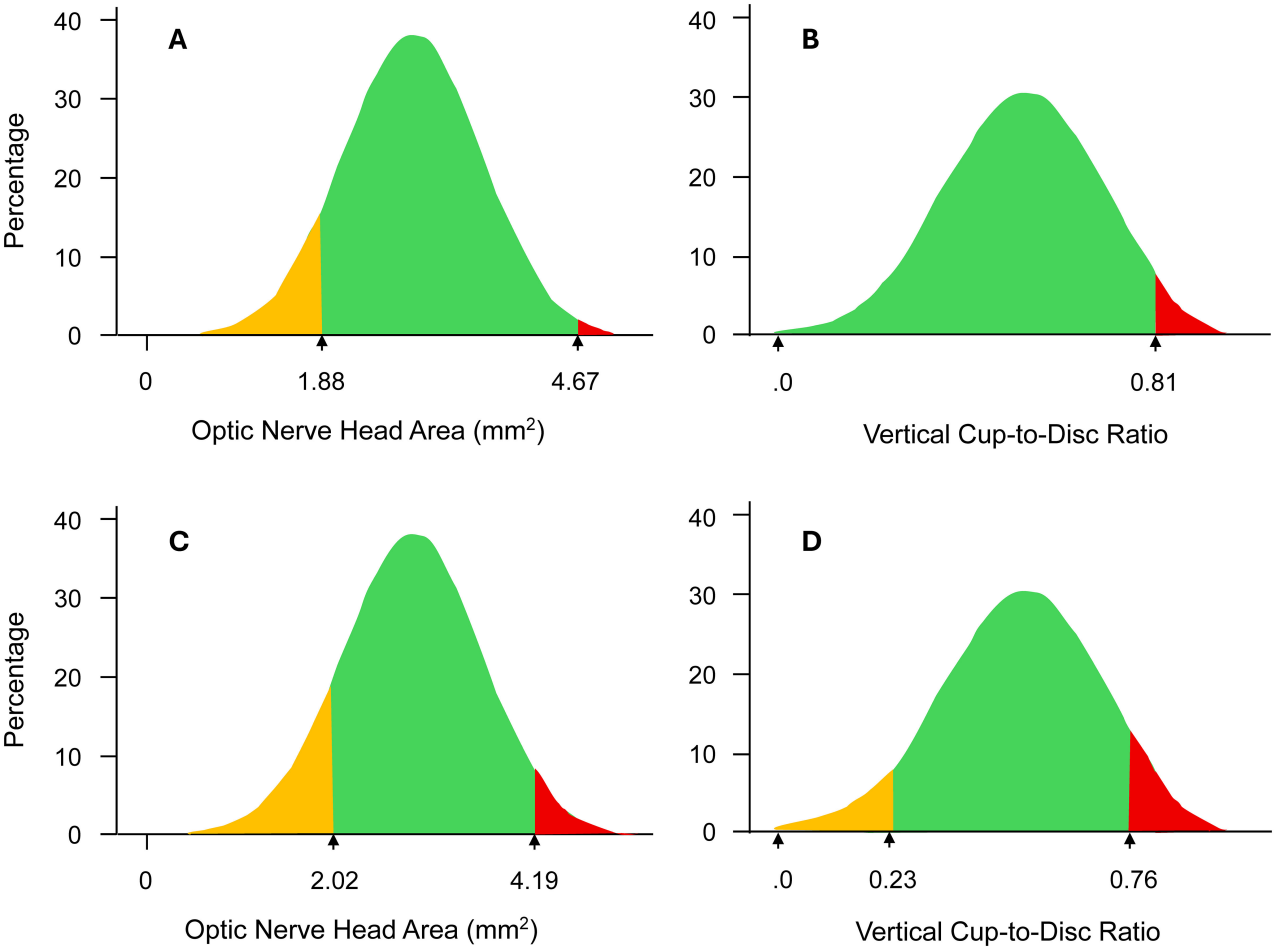
Normative distribution of optic nerve head area and vertical cup-to-disc ratio indicating the 2.5^th^ and 97.5^th^ percentiles **(A, B)** and the 5^th^ and 95^th^ percentiles **(C, D)**. Green In **(A, B)** represents 95% of measurements comprised between the 2.5^th^ and 97.5^th^ percentiles; in **(C, D)** it indicates 90% of measurements ranging between the 5^th^ and 95^th^ percentiles. Red indicates measurements greater than the 97.5^th^ percentiles in **(A, B)** and greater than the 95^th^ percentile in **(C, D)**. Orange area means 2.5% measurements thinner than the 2.5^th^ percentile **(A, B)** and 5% measurements thinner than the 5^th^ percentile **(C, D)**.

**Table 2** presents the normal distributions of optic disc parameters’ measurements regardless of age. Only data for the right eye is presented as no differences were observed in all measurements between fellow eyes. Median (IQR), lower or 2.5^th^ percentile, and upper limits or 97.5^th^ percentile were 2.70 (0.92) mm^2^, 1.88 and 4.67 mm^2^ for ODA; 2.03 (0.32) mm, 1.64 mm and 2.64 mm for ODVD; 1.70 (0.5) mm, 1.41 mm and 2.20 mm for ODHD; 0.57 (0.20), 0.18 and 0.81 for VCDR; 0.89 (0.81) mm^2^, 0.13 and 2.60 mm^2^ for ODCA; 1.74 (0.75) mm^2^, 0.85 and 3.22 mm^2^ for ODRA, respectively. Of all parameters, only ODRA differed significantly between women and men (p < 0.001 for medians and p = 0.002 for means), with women having a larger rim area than men. **Table 2** also presents the lower (5^th^ percentile) and upper (95^th^ percentile) limits of normality based on OCT classification: 2.02 and 4.19 mm^2^ (ODA), 1.64 and 2.51 mm (ODVD), 0.23 and 0.76 (VCDR), 0.11 and 2.33 mm^2^ (ODCA), and 1.69 and 3.01 mm^2^ (ODRA). The normal distribution curves with the 2.5^th^ and 97.5^th^ percentiles for ODA and VCDR are illustrated in **Figures 1A, B**, respectively, whereas the 5^th^ and 95^th^ percentiles are indicated in **Figures 1C, D**.

**Table 2:** Median and mean optic disc topographic measurements in all participants, men, and women.

Measurements	ODA	ODVD	ODHD	VCDR	ACDR	ODCA	ODRA
Mean	2.86 ± 0.73	2.06 ± 0.23	1.75 ± 0.22	0.54 ± 0.17	0.34 ± 0.18	1.03 ± 0.81	1.81 ± 0.65
2.5^th^ percentile	1.88	1.64	1.41	0.00	0.00	0.00	1.63
5^th^ percentile	2.02	1.73	1.43	0.23	0.04	0.11	1.69
Median (IQR)	2.70 (0.92)	2.03 (0.32)	1.70 (0.35)	0.57 (0.20)	0.34 (0.24)	0.89 (0.81)	1.74 (0.75)
95^th^ percentile	4.19	2.51	2.20	0.76	0.63	2.33	3.01
97.5^th^ percentile	4.67	2.64	2.24	0.81	0.67	2.60	3.22
Men median	2.62 (0.99)	1.98 (0.36)	1.70 (0.31)	0.59 (0.22)	0.38 (0.25)	0.99 (0.90)	1.60 (0.63)
Women median	2.80 (0.90)	2.04 (0.28)	1.73 (0.38)	0.55 (0.19)	0.33 (0.20)	0.85 (0.78)	1.82 (0.69)
Median p	0.34	0.18	0.36	0.17	0.048	0.18	<0.001
Men mean	2.86 ± 0.79	2.04 ± 0.23	1.74 ± 0.22	0.54 ± 0.18	0.37 ± 0.19	1.13 ± 1.01	1.68 ± 0.73
Women mean	2.88 ± 0.68	2.08 ± 0.23	1.76 ± 0.23	0.53 ± 0.15	0.33 ± 0.16	0.98 ± 0.63	1.92 ± 0.57
Mean p	0.89	0.26	0.37	0.73	0.06	0.16	0.002

ODA, optic disc area; ODVD, optic disc vertical diameter; ODHD, optic disc horizontal diameter; VCDR, vertical cup-to-disc ratio; ACDR, average cup-to-disc area ratio; ODCA, optic disc cup area; ODRA, optic disc rim area; IQR, interquartile range.

The normal distribution (2.5^th^, median, and 97.5^th^ percentiles) and the OCT classification (5^th^ and 95^th^ percentiles) stratified by age group are provided in **Table 3**. Medians of all parameters did not differ between age bins (all p > 0.05).

**Table 3:** Median and mean optic disc topographic measurements by age group.

Measurements	ODA	ODVD	ODHD	VCDR	ACDR	ODCA	ODRA
18–29 years							
2.5^th^ percentile	1.88	1.62	1.39	0.02	0.00	0.01	1.73
5^th^ percentile	2.00	1.66	1.44	0.19	0.04	0.10	1.74
Median (IQR)	2.82 (0.97)	2.04 (0.32)	1.73 (0.36)	0.55 (0.19)	0.33 (0.25)	0.89 (0.93)	1.87 (0.67)
95^th^ percentile	4.66	2.63	2.23	0.74	0.60	2.27	3.18
97.5^th^ percentile	4.75	2.69	2.26	0.76	0.63	2.58	3.34
Mean ± SD	2.93 ± 0.72	2.09 ± 0.24	1.77 ± 0.23	0.53 ± 0.16	0.34 ± 0.16	1.01 ± 0.64	1.88 ± 0.66
30–39 years							
2.5^th^ percentile	1.89	1.64	1.30	0.02	0.00	0.01	1.73
5^th^ percentile	1.92	1.79	1.41	0.18	0.04	0.09	1.78
Median (IQR)	2.60 (1.06)	1.98 (0.30)	1.66 (0.36)	0.56 (0.21)	0.32 (0.21)	0.85 (0.71)	1.84 (0.75)
95^th^ percentile	4.22	2.42	2.22	0.70	0.53	2.16	2.93
97.5^th^ percentile	4.53	2.05	2.25	0.92	0.81	2.71	3.87
Mean ± SD	2.82 ± 0.95	2.03 ± 0.20	1.74 ± 0.24	0.51 ± 0.17	0.30 ± 0.16	1.02 ± 1.29	1.91 (0.67)
40–49 years							
2.5^th^ percentile	1.91	1.68	1.42	0.00	0.00	0.00	1.68
5^th^ percentile	2.02	1.75	1.44	0.19	0.02	0.05	1.71
Median (IQR)	2.75 (0.82)	2.05 (0.32)	1.71 (0.29)	0.58 (0.22)	0.36 (0.27)	0.99 (0.84)	1.76 (0.73)
95^th^ percentile	4.48	2.50	2.15	0.86	0.66	2.14	2.45
97.5^th^ percentile	4.51	2.67	2.32	0.90	0.92	2.76	2.66
Mean ± SD	2.83 ± 0.63	2.07 ± 0.23	1.74 ± 0.21	0.55 ± 0.18	0.37 ± 0.21	1.06 ± 0.64	1.62 ± 0.59
≥50 years							
2.5^th^ percentile	1.82	1.53	1.36	0.06	0.02	0.06	1.65
5^th^ percentile	2.09	1.65	1.42	0.33	0.11	0.29	1.66
Median (IQR)	2.63 (0.97)	1.99 (0.34)	1.70 (0.41)	0.58 (0.25)	0.33 (0.26)	0.87 (0.84)	1.79 (0.77)
95^th^ percentile	3.95	2.41	2.16	0.77	0.67	2.47	3.17
97.5^th^ percentile	4.49	2.66	2.22	0.83	0.75	3.26	3.75
Mean ± SD	2.82 ± 0.61	2.04 ± 0.23	1.75 ± 0.22	0.56 ± 0.16	0.35 ± 0.17	1.05 ± 0.68	1.86 ± 0.69
Median p	0.37	0.39	0.53	0.41	0.54	0.60	0.11
Mean p	0.77	0.53	0.83	0.38	0.16	0.74	0.049

ODA, Optic disc area; ODVD, optic disc vertical diameter; ODHD, optic disc horizontal diameter; VCDR, vertical cup-to-disc ratio; ACDR, average cup-to-disc area ratio; ODCA, optic disc cup area; ODRA, optic disc rim area; IQR, interquartile range.

### 3.3 Correlation of ONH parameters with age

The results of the Spearman’s rank correlation of ONH parameters with age as well as the correlation among ONH parameters indicated that none of the ONH parameters measured in this study correlated with age (all p>0.05). ODA correlated positively with ODHD, VCDR, ACDR, ODCA, and ODRA (all p <0.001). ODRA correlated negatively with ODCA (p = 0.004), VCDR and ACDR (all p < 0.001) but positively with ODVD (p < 0.001).

## 4 Discussion

In this study, we evaluated the profile of ONH parameters acquired with spectral domain OCT in a nonglaucomatous black people from the DRC. Normative values for ONH parameters have been reported in some populations. In comparison with most previous studies, the particularity of our study is to have reported normative values in percentiles with IQR rather than mean with minimum and maximum values or lower and upper bounds of 95% confidence interval. Percentiles are more resistant to outliers and allow for a more accurate representation of data distribution, especially in skewed datasets. They also enable easy comparison across different groups and provide a standardized way to interpret data, making it simpler to understand relative standing of an observation within a dataset.

**Table 4** provides normative values of ONH parameters from studies in different African populations. Both the median and mean in our study are greater than reported by other OCT-based studies within and outside SSA regardless of whether Topcon OCT (8, 11) or Cirrus OCT platform (7, 9, 10) was used. Our mean ODA was also greater than HRT-based value of 2.56 mm^2^ reported previously in the same setting (12). A planimetry-based mean ODA comparable to our OCT-based mean was reported in black people in the Baltimore Eye Survey (3). A careful read of the information contained in **Table 4** reveals the following facts: 1) There is variability in the ONH size across different populations; 2) Our observation and that of Krueger et al. (12) indicate a larger ONH in Congolese than in other SSA populations. There are several possible reasons for the variability of ODA between studies. First, SSA is highly ethnically and genetically diverse, and both are important determinants of optic disc size and morphology (18). Additional plausible reasons include differences in sample composition across studies and differences in the accuracy of segmentation algorithms on different OCT platforms, and between OCT and HRT.

**Table 4:** Comparison of reference ONH topographic measurements across studies in people of people of African descent.

Study	Country	Study type	N	Mean age	Measurement method	ODA*	ODRA*	ODVD*	ODHD*	VCDR*	ACDR*	ODCA*
Present study	DRC	HBS	263	35.3	OCT (Topcon)	2.70 [2.86]	1.92 [1.81]	2.11 [2.11]	1.79 [1.77]	0.54 [0.57]	0.34 [0.33]	1.06 [0.93]
Gessesse et al. (7)	Ethiopia	HBS	165	40.8	OCT (Cirrus)	[2.01]	[1.45]	[1.50]	–	[0.46]	[0.48]	–
Mba et al. (8)	Gabon	HBS	292	43.2	OCT (Topcon)	[2.57]	[1.42]	–	–	[0.61]	–	[1.11]
Nelson-Ayifah et al. (9)	Ghana	HBS	338	61.9	OCT (Cirrus)	[2.05]	[1.52]	–	–	[0.44]	[0.47]	–
Ocansey et al. (10)	Ghana	HBS	100	40.7	OCT (Cirrus)	[2.08]	[1.48]	–	–	[0.47]	[0.49]	–
Awe et al. (11)	Nigeria	HBS	88	52.6	OCT (Topcon)	[2.54]	–	[1.93]	[1.87]	–	–	–
Krueger et al. (12)	DRC	HBS	207	45.6	HRT	[2.56]	[1.91]	–	–	–	–	–
Varma et al. (3)	USA	PBS	2210		Planimetry	[2.94]	[1.90]	–	–	[0.56]	–	[1.04]

*Values not in brackets are medians, those in brackets are means; HBS, hospital-based study; PBS, population-based study; ODVD, optic disc vertical diameter; ODHD, optic disc horizontal diameter; VCDR, vertical cup-to-disc ratio; CDR, average cup-to-disc area ratio; ODCA, optic disc cup area; ODRA, optic disc rim area.

The ODCA in our sample was also larger than reported in the studies listed in **Table 4** regardless of the method used, except for comparable mean ODCA reported among Gabonese (8) and among black people in the Baltimore Eye Survey (3). Thus, our study corroborates that larger ONH have wide excavations and vice versa. On a practical level, this observation requires the clinician to be careful not to incorrectly label a normal subject having a large ONH with a wide excavation as glaucomatous or, conversely, to miss diagnosing a glaucomatous subject with a small excavation on a small ONH (19, 20). In both scenarios, the therapeutic implications are not negligible.

The 97.5^th^ percentile for VCDR in this population was 0.81. Population-based studies in SSA have reported values of 0.73 in Ghana (4), 0.75 in Nigeria (21), 0.70 in Kenya (22), and 0.72 in Tanzania (23) and South Africa (24). The 97.5^th^ percentile for VCDR is an important parameter in the normal population because it is the cutoff recommended by the International Society for Geographic and Epidemiologic Ophthalmology (ISGEO) for the diagnosis of glaucoma in epidemiologic studies in light of racial/ethnic variations in optic disc morphology (17). It is important to note that VCDR estimation in these studies was made with fundus biomicroscopy, planimetry or optic disc photograph evaluation. While a normal higher VCDR can be a risk factor for glaucoma, it’s not always indicative of the disease, especially in individuals with larger optic discs., It is therefore crucial to consider the overall optic disc size when interpreting VCDR regardless of the classification used, as a larger disc can naturally accommodate a larger cup and a larger VCDR while still healthy, whereas it should raise suspicion for glaucoma and warrant further investigation in the presence of other risk factors (e.g. a small optic disc, vertical elongation of the cup or a significant increase of the VCDR over time).

We found no difference in all ONH between men and women, expect for significantly greater median and mean ODRA in women than men. In previous studies, this comparison had produced varying findings. Results like ours have been reported in a Ghanaian hospital-based study (10). In the people of African descent who participated in the Baltimore Eye Survey, ODA was larger in men than in women, ODRA was similar in both sexes, and VCDR followed the same trend as ODA (3). To sum up, while some research points to differences in optic disc size between men and women, these findings are inconclusive. The individual variation in optic disc size and the importance of considering other diagnostic factors imply that it is not recommended to focus solely on optic disc size based on gender for diagnosing or evaluating conditions like glaucoma.

None of the optic disc topographic parameters correlated with age in this series; this relationship has been investigated in several studies. Contrary to our finding, ODRA reduction with increasing age has been reported in normal subjects in some studies (1, 10, 25), likely reflecting the physiological age-related loss of retinal ganglion cell (RGC) axons. Taken together, these observations indicate that the ODRA-age relationship is inconsistent. Reasons include: 1) Variations in measurement techniques as different studies may use different methods to measure the ODRA (i.e. OCT, confocal scanning laser ophthalmoscopy, planimetry), which have different strengths and limitations that can lead to discrepancies in the results; 2) Study design limitations as cross-sectional studies may not accurately capture age-related changes, while longitudinal studies are often limited by sample size and follow-up periods; 3) The specific population studied (e.g., ethnicity, refractive error) can also influence the findings; 4) The complexity of aging process that can affect various components of the optic nerve head, including both neural and non-neural tissue so that the interplay between these changes can influence the overall ODRA measurement; and 5) Significant individual variability in the rate and pattern of age-related changes in the ODRA. Some individuals may experience more significant thinning than others, even at the same age. The relationship between ODA and age has also been examined in SSA. In contrast to our finding that ODA does not change significantly with age, a decline in ODA with increasing age has been reported among Ghanaians (10) and Nigerians (11). The significant relationship observed among optic disc parameters, notably between ODA and ODCA, was not surprising and confirms the results of previous studies. Clinically, this relationship is often assessed through VCDR or ACDR. A higher VCDR generally indicates a larger cup relative to the disc, which can be a sign of glaucoma. However, this relationship is not linear since a high VCDR on a small disc might be more concerning than the same ratio on a large disc. Thus, interpretation of VCDR needs to take into consideration the overall optic disc size. It is also important to note that even though larger optic discs tend to have larger cups, large disc may also have a high VCDR without being glaucomatous, while a small disc may have a low VCDR but still be glaucomatous. From the clinical standpoint, evaluating the CDR is of utmost importance in glaucoma screening, but it is not the only factor to consider. Other factors, such as VCDR asymmetry between the two eyes, the appearance of the neuroretinal rim, nerve fiber layer defects, and visual field testing, are also important.

## 5 Conclusion

We established normative values for ONH parameters in a Congolese population using percentile-based limits of normality, providing a reference framework for clinical interpretation. ODA was larger than values reported in other SSA populations and showed no association with age, while the optic disc rim area was not correlated with either age or IOP. These observations underscore population-specific characteristics of the ONH that are relevant for diagnosis and disease monitoring. Although derived from OCT measurements, these normative data contribute more broadly to improved interpretation of ONH structure and support more accurate diagnostic decision-making, risk stratification, longitudinal assessment, ultimately contributing to improved clinical management of Congolese patients regardless of the imaging platform employed. Advances in posterior segment imaging technologies continue to evolve and to enhance our understanding of the ONH, which remains central to disease detection and progression assessment, irrespective of the specific modality employed.

## Data Availability

The raw data supporting the conclusions of this article will be made available by the authors, without undue reservation.
